# Analysis of Professionalism Themes Raised in Evaluations of Faculty

**DOI:** 10.1001/jamanetworkopen.2022.21613

**Published:** 2022-07-13

**Authors:** Janae K. Heath, Caitlin B. Clancy, C. Jessica Dine, William J. S. Pluta, Jennifer R. Kogan

**Affiliations:** 1 Department of Medicine, Perelman School of Medicine, University of Pennsylvania, Philadelphia

## Abstract

This qualitative study examines unprofessional behavior by medical center faculty according to narrative evaluations.

## Introduction

Unprofessional behavior by medical faculty negatively impacts the clinical learning environment and learner outcomes.^1,2^ One method to identify faculty professionalism lapses is through review of learners’ narrative evaluations,^3^ yet it remains unclear whether the diverse domains of unprofessionalism (spanning patient, learner, and interprofessional interactions)^4^ are captured in these evaluations. This study aimed to classify domains and frequencies of unprofessional behavior referenced in narrative evaluations of faculty.

## Methods

We performed a qualitative study of narrative evaluations of faculty at a single, large academic medical center, using a random sample of deidentified faculty evaluations written by medical students, residents, and fellows from July 1, 2017, to December 31, 2019. The study was deemed exempt by the University of Pennsylvania’s institutional review board because it was believed to pose minimal risk to study participants. This study followed the Standards for Reporting Qualitative Research (SRQR) reporting guideline.

We conducted a summative content analysis of comments, using the constant comparative method, to identify and classify professional lapses. An initial codebook was developed using prior classifications of unprofessional behavior,^1,4^ with iterative revisions made by consensus at prespecified coding intervals. Comments were coded for references to unprofessional behavior and the domain(s) observed in each comment. Three coders (J.K.H., C.B.C., and J.R.K.) jointly coded an initial set of 100 comments, then independently coded 100 additional comments using Excel, version 16.16.1 (Microsoft Corporation), with subsequent group consensus. Ten percent of comments from 2017 to 2018 were triple-coded to ensure greater than 80% agreement by κ scores, and the remaining comments were coded with 5% triple-coding. We performed summary statistics to quantify the professionalism themes. Analyses were conducted from October 2021 to February 2022.

## Results

A total of 15 432 evaluations were analyzed (32% of 48 384 learner evaluations) (Table 1). A total of 1004 professionalism lapses were identified within 735 evaluations with references to unprofessional behavior (5% of total evaluations). Interrater reliability was high (κ = 0.71-1.0; mean [SD], 0.87 [0.15]). Table 2 lists the themes observed. A total of 837 professionalism lapses [83%] pertained to disrespectful interactions with colleagues, coworkers, and students, whereas 86 references [9%] were related to observed interactions with patients and their families. The most common theme was emotional disrespect of the learner (279 [28%]), followed by failure to include, acknowledge, and/or validate learners (114 [11%]). Sexual harassment (18 [2%]), harassment based on race or ethnicity (8 [0.8%]) or sexual orientation (3 [0.3%]), and physical disrespect (7 [0.7%]) were less common.

**Table 1.** Demographic Characteristics of Faculty in Cohort of Evaluations Used in Analysis

Characteristic	No. (%)
Total evaluations	15 432 (100)
Evaluations with any professional lapse identified	735 (5)
Evaluations completed, by level of trainee	
Residents and fellows	11 684 (76)
Medical students	3700 (24)
Missing	48 (0)
Total faculty	2470 (100)
Sex	
Female	1023 (41)
Male	1304 (53)
Undisclosed or missing	143 (6)
Race and ethnicity	
Asian	443 (18)
Black	90 (4)
Hispanic	62 (3)
White	1662 (67)
Multiple	40 (2)
Undisclosed or missing	173(7)
Department	
Anesthesiology and critical care	212 (9)
Dermatology	57 (2)
Emergency medicine	81 (3)
Family medicine	39 (2)
Genetics	1 (0)
Medicine	616 (25)
Neurology	116 (5)
Neurosurgery	20 (1)
Obstetrics and gynecology	86 (3)
Ophthalmology	33 (1)
Orthopedic surgery	49 (2)
Otorhinolaryngology	38 (2)
Pathology	84 (3)
Pediatrics	463 (19)
Physical medicine and rehabilitation	26 (1)
Psychiatry	121 (5)
Radiation oncology	36 (1)
Radiology	160 (6)
Surgery	154 (6)
Nonclinical department^a^	4 (0)
Undisclosed or missing	74 (3)

^a^ Nonclinical departments include biostatistics and epidemiology, cell and developmental biology, and medical ethics and health policy.

**Table 2.** Professionalism Themes and Subthemes in Faculty Evaluations Written by Medical Students, Residents, and Fellows^a^

Professionalism lapse themes and subthemes	No. (%)
Interactions with patients and/or patients’ families	
Not respecting patients’ or families’ decisions, wishes, or needs	3 (0)
Dismissive of patient or family concerns	8 (1)
Failure to take ownership of patient, family issues, complaints, and concerns	15 (2)
Using inappropriate language with a patient or family	6 (1)
Treating the patient as a disease carrier rather than a person	0
Being disrespectful to stigmatized populations	9 (1)
Using inappropriate humor/comments with or about patients and families	5 (1)
Nonspecific disrespect toward patients and families	40 (4)
Interactions with colleagues, coworkers, and learners	
Using inappropriate language (cursing)	14 (1)
Using inappropriate humor (sarcasm, off color humor)	18 (2)
Inappropriate discussions about personal information	5 (0)
Sexual harassment (denied opportunities based on sex, exchange of sexual favors, unwanted sexual advances, offensive remarks	18 (2)
Racial and/or ethnic harassment (denied opportunities based on race, offensive remarks)	8 (1)
Sexual orientation harassment (denied opportunities based on sexual orientation, offensive remarks)	3 (0)
Physical disrespect (physical harm, lack of physical safety)	7 (1)
Emotional disrespect (belittling, bullying lack of psychological safety, criticizing others), toxic learning environment, and/or malignant learning environment (including judgmental learning environment, intolerance of mistakes)	279 (28)
Failure to include and/or acknowledge and/or validate the learner(s)	114 (11)
Failure to include and/or acknowledge and/or validate the members of the interprofessional team	14 (1)
Assigning or expecting inappropriate responsibilities to the learners’ capabilities	19 (2)
Disrespect for individuals’ role (asked to do personal services)	4 (0)
Not providing equal opportunities (playing favorites)	18 (2)
Not respecting time, tardiness (physical presence)	68 (7)
Not responding to emails or telephone communication	8 (1)
Unavailable to trainee	101 (10)
Nonspecific comment about an unwelcome or uncomfortable learning environment	78 (8)
Nonspecific disrespect with colleagues, coworkers, or learners	61 (6)
Honesty, responsibility, integrity issues	8 (1)
Nonspecific professionalism concern not captured elsewhere	73 (7)

^a^ Evaluations included a numeric rating for the statement “Rate the overall quality of the teaching” followed by a free-text question (labeled “Comments”).

## Discussion

Narrative evaluations of faculty contained references to unprofessional behavior related to emotional disrespect of learners and unhealthy learning environments. However, references to sexual harassment, mistreatment based on race or ethnicity or sexual orientation, and physical disrespect were rare and lower than institutional and national survey prevalence.^5^ This finding could indicate learner reluctance to document these behaviors in standard evaluations because of confidentiality and retribution concerns and/or preference for alternate formal or informal reporting processes.

Faculty professionalism lapses are challenging to identify for multiple reasons, including misperceptions of the seriousness of the behavior, uncertainty with reporting processes, and fear of retaliation.^3,6^ Our study demonstrated that review of narrative evaluations of faculty may be useful in identifying concerns about emotional disrespect and poor learning environments but probably does not capture behavior related to sexual harassment, race or ethnicity– or sexual orientation–based mistreatment, or physical disrespect.

Beyond the learner findings, references to patient-related unprofessional behavior were also infrequent, raising the possibility that learners think that evaluations should focus on teaching skills. This finding further suggests a need for multiple strategies for identifying unprofessional faculty behavior, possibly including a designated prompt or avenue for patient-related issues.

Study limitations include the single-center cohort and the inability to determine associations with faculty and trainee characteristics. Nevertheless, our study offers novel information about domains of unprofessional behavior referenced in faculty evaluations. Research determining optimal strategies for identification of faculty professionalism lapses, as well as understanding trainee behaviors and preferences for reporting, is critical to uphold a safe clinical learning environment.
